# Exogenous Calcium Alleviates Oxidative Stress Caused by Salt Stress in Peanut Seedling Roots by Regulating the Antioxidant Enzyme System and Flavonoid Biosynthesis

**DOI:** 10.3390/antiox13020233

**Published:** 2024-02-14

**Authors:** Yan Gao, Xuan Dong, Rongjin Wang, Fei Hao, Hui Zhang, Yongyong Zhang, Guolin Lin

**Affiliations:** College of Land and Environment, Shenyang Agricultural University, No. 120 Dongling Road, Shenhe District, Shenyang 110866, China; gaoyan2915@163.com (Y.G.); 2019200115@stu.syau.edu.cn (X.D.); 2021220451@stu.syau.edu.cn (R.W.); haofei@stu.syau.edu.cn (F.H.); huizhang@syau.edu.cn (H.Z.); yongyongzhang@syau.edu.cn (Y.Z.)

**Keywords:** exogenous calcium, metabolomic, transcriptomic, flavonoids, salt stress, antioxidant, reactive oxygen species

## Abstract

Soil salinity is one of the adversity stresses plants face, and antioxidant defense mechanisms play an essential role in plant resistance. We investigated the effects of exogenous calcium on the antioxidant defense system in peanut seedling roots that are under salt stress by using indices including the transcriptome and absolute quantitative metabolome of flavonoids. Under salt stress conditions, the antioxidant defense capacity of enzymatic systems was weakened and the antioxidant capacity of the linked AsA-GSH cycle was effectively inhibited. In contrast, the ascorbate biosynthesis pathway and its upstream glycolysis metabolism pathway became active, which stimulated shikimate biosynthesis and the downstream phenylpropanoid metabolism pathway, resulting in an increased accumulation of flavonoids, which, as one of the antioxidants in the non-enzymatic system, provide hydroxyl radicals to scavenge the excess reactive oxygen species and maintain the plant’s vital activities. However, the addition of exogenous calcium caused changes in the antioxidant defense system in the peanut root system. The activity of antioxidant enzymes and the antioxidant capacity of the AsA-GSH cycle were enhanced. Therefore, glycolysis and phenylpropanoid metabolism do not exert antioxidant function, and flavonoids were no longer synthesized. In addition, antioxidant enzymes and the AsA-GSH cycle showed a trade-off relationship with sugars and flavonoids.

## 1. Introduction

Soil salinization is one of the major abiotic stress factors limiting crop production [1]. Based on relevant statistics, salinized soil has reached an area of 8.31 × 10^8^ ha worldwide [2]. Approximately 20% of cultivated land and 50% of irrigated soils are salinized [3]. Excessive soil salinity can adversely affect plant growth, quality, and yield [4]. Ion toxicity and osmotic stress are triggered when plants are subjected to salt stress, which is accompanied by secondary oxidative stress and the accumulation of reactive oxygen species (ROS), which are highly reactive and toxic and contribute to the damage to proteins, lipids, carbohydrates, and DNA [5]. These compounds cause cellular oxidative damage, reduced photosynthesis, and reduced root vigor and ultimately impair plant growth and development [3,6].

Plants can regulate antioxidant defense mechanisms to scavenge the excess reactive oxygen species through two ways, thereby improving adaptation to salt stress [7]. In the enzymatic antioxidant system, SOD, CAT, and various POD catalyze the reduction of reactive oxygen species. In addition, AsA and GSH can serve as important hydrogen-donating substrates in the AsA-GSH cycle [8], which work in synergy with antioxidant enzymes to scavenge reactive oxygen species through regeneration by ascorbate peroxidase, monodehydroascorbate reductase, dehydroascorbate reductase, and glutathione reductase [5]. It has been shown that up-regulation or overexpression of enzymes in the AsA-GSH cycle and increased levels of AsA and GSH lead to better tolerance in plants regarding abiotic stresses by decreasing ROS [9,10,11,12]. The second way is through metabolic reprogramming, i.e., the ratio of primary and secondary metabolisms, to improve its adaptation to adversity stress, including salt stress [13]. Moreover, increased flavonoid content has been shown to scavenge free radicals generated by adversity, mitigate membrane lipid peroxidation, and act as protectors of cell walls and membranes, thereby improving plant resistance to both biotic and abiotic stresses [14]. Phytochemicals such as flavonoids are widely distributed in plants as important secondary metabolites. Plants can improve salt tolerance by regulating the composition of membrane lipids at the cellular level [15]. Recently, it has been suggested that sugar may act as a true ROS scavenger in plants [16]. Stress-induced ROS scavengers or products of repair enzymes can utilize sugar, at high or low concentrations. Additionally, soluble sugars accumulate in plants under abiotic and biotic stress conditions involving oxidative stress, suggesting that sugars play a key role in ROS accumulation during stress [17,18]. Fructans are considered to be more than just reserve water-soluble oligosaccharides and polysaccharides that act directly as ROS scavengers near the vesicular membrane [17]. Further, fructan sugars can scavenge (▪OH) hydroxyl radicals through vesicular oxidases and peroxidases, thereby preventing lipid peroxidation [19]. Gluconeogenesis is downstream of AsA biosynthesis. Hence, sugar metabolism links antioxidant enzymes to the AsA-GSH defense system and C6-C3-like secondary metabolism, constituting plants’ main framework for antioxidant protection [4]. Furthermore, it is essential to point out that the balance pattern among the three under salt stress may differ based on the species of plants and the time and space of stress.

Calcium ions act as second messengers involved in plant salt stress responses. Under salt stress, which may result in a transient increase in [Ca^2+^]_cyt_, mechanosensitive plasma membrane Ca^2+^ permeability channels detect changes in osmotic potential [20]. Osmotic and ionic stress increase calcium ion concentration in the cytoplasm [21]. Abiotic stress activates calcium-binding proteins, which detect adversity signals [22]. Exogenous calcium can enhance plants’ salt stress resistance. Previous studies have shown that calcium enhances plant photosynthesis, promotes seed germination, and increases plant water under salt stress [23]. Exogenous calcium can alleviate secondary oxidative stress caused by salt stress by increasing plant antioxidant enzyme activities and reducing membrane lipid peroxidation [24]. By restoring the ionic and osmotic balance, exogenous calcium ions improve peanut tolerance to salt stress [4,24]. However, recent studies have shown that exogenous calcium can enhance wheat adaptation to salt stress by increasing the activity or content of enzymatic and non-enzymatic antioxidants [25].

Peanut (*Arachis hypogaea* L.) is a common oilseed crop grown in over 100 countries worldwide [26]. Salt stress is often associated with cultivating peanuts. However, peanuts are calcium-loving crops [27,28,29,30]; calcium fertilizer application can significantly increase peanut emergence rate, promote peanut growth, and improve yield and quality. In the meantime, exogenous calcium can alleviate salt stress [31]. Previous studies of the same kind have focused on the effects of exogenous calcium on antioxidant enzyme activity, cation content, agronomic traits, and yield of peanuts. Conversely, few studies have examined plant antioxidant defense mechanisms under adverse conditions. In this study, we combined plant physiology, transcriptomics, and metabolomics to investigate the effects of exogenous calcium on antioxidant enzymes, the AsA-GSH cycling system, sugar metabolism, and flavonoid biosynthesis pathways in peanut roots under salt stress. These results help us better understand how exogenous calcium responds to oxidative stress, thus alleviating salt stress. It also provides a theoretical basis for applying exogenous calcium in salinized soils.

## 2. Materials and Methods

### 2.1. Experimental Location and Experimental Materials

Peanut seeds (*Arachis hypogaea* L. cv. Haihua 1, salt-sensitive variety) were obtained from Qilu Seed Mall, Jinan, China. The peanut seeds with intact seed coats were soaked in an electrically heated thermostatic water bath at 35 degrees Celsius for eight hours. Seeds were placed in trays and incubated for three days at 29 degrees Celsius without light after rapid absorption and swelling of water. Seedlings with uniform growth were inserted into germination pots, which were then placed in a thermostatic incubator at 25 degrees Celsius and cultivated for four days without light for subsequent experiments.

### 2.2. Experimental Design

This experiment was conducted by aqueous solution culture in May 2022 at the National Engineering Research Center for Efficient Utilization of Soil Fertilizer, College of Land and Environment, Shenyang Agricultural University, Shenyang, China. A total of four treatments were set up in this experiment, namely (1) distilled water (CK), (2) NaCl treatment (Na), (3) CaCl_2_ treatment (Ca), and (4) NaCl+CaCl_2_ treatment (Na_Ca), in which NaCl was at a concentration of 150 mmol/L, which is high salt stress, and CaCl_2_ was at 15 mmol/L. Each treatment was set up with three replications, randomized zonal arrangement, a total of 12 pots, 15 peanut seedlings per pot, and added treatment solution and incubated at 25 °C under no light condition for 48 h.

### 2.3. Root Physiological Analysis

The peanut root system was separated and immediately placed in liquid nitrogen for rapid freezing, after which it was stored in a −80 °C refrigerator for subsequent determination of the indices. The activity of antioxidant enzymes in the root system (0.5 g) was determined spectrophotometrically [32]. Superoxide dismutase (SOD) activity was measured at 560 nm based on NBT photochemical reduction. Peroxidase (POD) activity was measured at 470 nm based on the absorbance of guaiacol. The absorbance values of the samples were measured at 240 nm, and catalase (CAT) activity was calculated by converting the rate of catalase consumption per unit time. Malondialdehyde (MDA) content was determined based on the content of thiobarbituric acid reactive substances (TBARSs) in root samples (nmol/g; extinction coefficient; 155 mMcm^−1^; [33]). The contents of H_2_O_2_, O^2−^, and soluble sugars in the root system were determined according to Wang et al. [33]. Root vigor was determined by the tetrazolium (2,3,5-triphenyl tetrazolium chloride, TTC) reduction method [34].

### 2.4. Transcriptome Data Analysis

Total RNA from 12 samples was subjected to transcriptome sequencing, and library construction was performed on qualified RNA. The RNA samples were sequenced and analyzed by Metware Biotechnology Co., Ltd. (Wuhan, China) using the Illumina HiSeq 2500 (Illumina, Inc, San Diego, CA, USA) sequencing platform. Raw sequencing reads were filtered by FASTP and mapped to the peanut genome using HISAT2 with default parameters. Differentially expressed genes (defined as a fold change in expression ≥ 2 or a fold change ≤ 0.5, *p* < 0.05) were analyzed using DESeq2. Gene functional annotation and pathway analysis were performed based on two databases: the GO (Gene Ontology) and KO (KEGG Ortholog database).

### 2.5. Metabolomic Data Analysis

UPLC-MS/MS analyzed the flavonoid metabolites of peanuts, and the flavonoid metabolites were extracted and identified by Metware Biotechnology Ltd. (Wuhan, China). The root samples were vacuum freeze-dried in a freeze-dryer (Scientz-100F, Zhejiang, China) and then ground for 1.5 min at 30 Hz to powder form using a ball mill (MM400, Retach, Germany). A total of 20 mg of the powder was weighed into 500 μL of 70% methanol aqueous solution (*v*/*v*) and mixed thoroughly using a multi-tube vortex shaker (MIX-200, Shanghai, China). The homogenate was ultrasonicated using an ultrasonic cleaner (KQ5200E, Kunshan, China) for 30 min before centrifugation (12,000 r/min) for 5 min at 4 °C. The supernatant was aspirated, and the sample was filtered through a 0.22 mm filter membrane and stored in the injection bottle.

Ultra-performance liquid chromatography (UPLC) was performed using a QTRAP6500+ instrument, Metware Biotechnology Co., Ltd. (Wuhan, China) from SCIEX equipped with a Waters ACQUITY UPLC HSS T3 C18 column (1.8 μm, 100 mm × 2.1 mm i.d.). The mobile phase consisted of ultrapure water with 0.05% formic acid (solvent A) and acetonitrile with 0.05% formic acid (solvent B). The elution gradients were 90:10 (*v*/*v*) for 0 min A/B, 80:20 (*v*/*v*) for 1 min A/B, 30:70 (*v*/*v*) for 9 min A/B, 5:95 (*v*/*v*) for 12.5 min A/B, 5:95 (*v*/*v*) for 13.5 min A/B, 90:10 (*v*/*v*) for 13.6 min A/B, and 15 min A/B was 90:10 (*v*/*v*). The column temperature was set at 40 °C, the flow rate was 0.35 mL/min, and the injection volume was 2 μL.

Tandem mass spectrometry (MS/MS) analysis was performed using the SCIEX 6500+ QTRAP, Metware Biotechnology Co., Ltd. (Wuhan, China). The air curtain gas (CUR) was set to 35 psi. In the Q-Trap 6500+, Metware Biotechnology Co., Ltd. (Wuhan, China) each ion pair was scanned for detection based on the optimized de-cluster voltage (DP) and collision energy (CE). Qualitative data analysis was based on the secondary spectral information in the MWDB (Metware Database) constructed by Metware Biotechnology Co., Ltd. (Wuhan, China), and flavonoids with variable projection importance (VIP) ≥ 1 and fold change (FC) > 2 were defined as differentially accumulated metabolites (DAMs). These flavonoids were analyzed for pathway enrichment using the Kyoto Encyclopedia of Genes and Genomes (KEGG) and Plant Metabolic Network (PMN) databases.

### 2.6. Real-Time Quantitative Polymerase Chain Reaction (RT-qPCR) Analysis

Total RNA was extracted and purified from peanut roots using the RNAiso Pure Plant Kit (TAKARA, Beijing, China) and then examined for total RNA by ultra-micro UV spectrophotometer (IMPLEN-N50, Thermo Scientific, USA) and 2% agarose gel electrophoresis (D50-UVIPURE, UVITE, UK; EPS300, Tanon, China) to check the quantity and quality of total RNA. Then, the total RNA was converted to complementary DNA (cDNA) by the GoScriptTM Reverse Transcription kit (Promega, USA) for subsequent RT-qPCR analysis. Primers were designed by Primer 3.0 and Primer 5.0 software, and the target gene sequences were found on NCBI’s GeneBank. RT-qPCR analyses were performed in the same manner as described in WEI [35], and the 2^−ΔΔCT^ calculated results [36] were obtained with this method with three replicates per treatment.

### 2.7. Data Analysis

The experimental data from the three independent biological replicate samples were analyzed using one-way analysis of variance (ANOVA), and the data were expressed as mean ± standard deviation (SD) of three replicates. Significant differences were determined by Duncan’s multiple range test (*p* < 0.05) using SPSS 22.0 statistical software (SPSS Inc., Chicago, IL, USA) [6]. Plots were created using online software (https://cloud.metware.cn/#/tools/tool-list , accessed on 1 June 2023) and in OriginPro 2021 (OriginLab, Northampton, MA, USA), and heat maps were plotted with TBtools. Log10 transformations were used to standardize the data in PCA.

## 3. Results

### 3.1. Effects of Exogenous Calcium on Physiological Indices of Peanut Root System under Salt Stress

**Figure 1:**
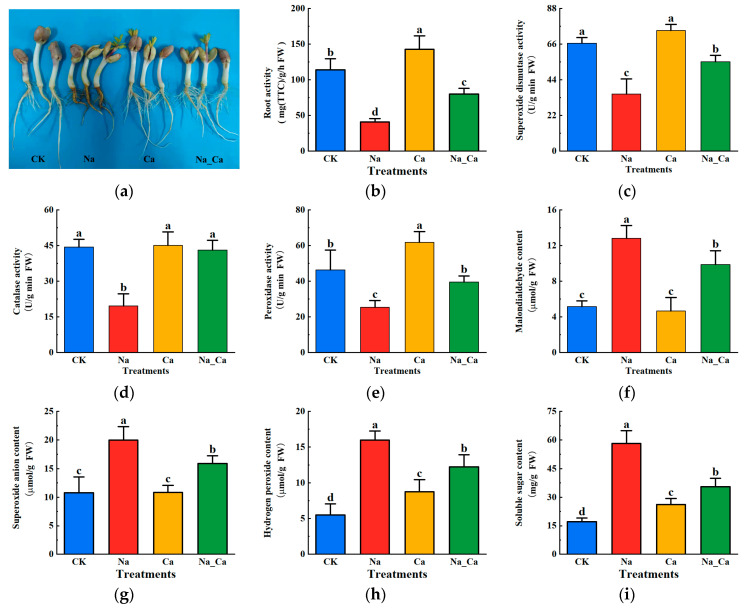
Changes in physiological indices of peanut roots under different treatments. (**a**) Phenotype of peanut roots under different treatments. (**b**) Root activity. (**c**) Superoxide dismutase, SOD. (**d**) Catalase, CAT. (**e**) Peroxidase, POD. (**f**) Malondialdehyde, MDA. (**g**) Superoxide anion, O^2−^. (**h**) Hydrogen peroxide, H2O2. (**i**) Soluble sugar. Different lowercase letters indicate differences between different treatments (*p* < 0.05).

In order to investigate the effects of exogenous calcium on peanut root phenotypes under salt stress, we took photographs of root growth and measured root vigor. And peanut root growth was inhibited under salt stress, while adding exogenous calcium significantly improved root growth (Figure 1a). The trends of root vigor under different treatments were consistent with the growth phenotype (Figure 1b). Compared to the CK treatment, the Ca treatment significantly increased root vigor by 25.26%, and the Na treatment significantly decreased root vigor by 64.18%. Root vigor significantly increased by 96.11% (*p* < 0.05) with Na_Ca treatment compared to that with Na treatment. The next step was to determine several physiological phenotypic indicators, including antioxidant enzyme activity (Figure 1c–e), MDA content (Figure 1f), and indicators reflecting oxidative stress, including O^2−^ and H_2_O_2_ (Figure 1g,h). The amount of soluble sugar was also determined (Figure 1i).

Antioxidant enzymes in plants are crucial in preventing oxidative stress damage to cells. As shown in Figure 1c–e, the trends of three antioxidant enzyme activities were the same under different treatments, and root growth was significantly better with exogenous calcium than that with CK treatment. The antioxidant enzyme activities also increased to varying degrees. SOD, CAT, and POD activities were significantly reduced by 34.47%, 55.61%, and 45.10% (*p* < 0.05) with Na treatment compared to that with CK treatment, respectively. Whereas, SOD, CAT, and POD activities were significantly increased by 56.04%, 119.04%, and 55.80% (*p* < 0.05) with Na_Ca treatment as compared to that with Na treatment, respectively.

MDA is an indicator of abiotic stress on plant cell membranes and responds to the level of abiotic stress on the plant. As can be seen from Figure 1f, MDA levels changed in an opposite direction to that of antioxidant enzyme activities in all four treatments. When Ca treatment was compared with CK treatment, the MDA content with Ca treatment was slightly, but not significantly, higher (*p* < 0.05), whereas Na treatment showed a significant increase of 147.49%. The MDA content with Na_Ca treatment was significantly reduced by 22.93% (*p* < 0.05) compared to that with Na treatment. The O^2−^ and H_2_O_2_ change trends were similar under different treatment conditions, as shown in Figure 1g,h. Na treatment significantly increased the O^2−^ and H_2_O_2_ contents by 85.53% and 190.91% (*p* < 0.05), respectively, compared to CK treatment. Compared to Na treatment, Na_Ca treatment significantly reduced O^2−^ and H_2_O_2_ contents by 20.50% and 23.50%, respectively (*p* < 0.05).

Soluble sugars are organic solutes accumulated by plants under adverse conditions that participate in osmoregulation and are used to maintain osmotic pressure balance, thereby reducing membrane damage caused by stress and preventing the denaturation of intracellular enzymes to maintain normal physiological functions. Under different treatments, the trend of soluble sugars was identical to that of malondialdehyde (Figure 1i). It was found that the soluble sugar content was maximized and significantly increased by 240.87% (*p* < 0.05) with Na treatment compared to that with CK treatment. Ca treatment also promoted the increase in soluble sugar content, significantly increasing by 53.51% (*p* < 0.05) compared to that with CK treatment. Compared to Na treatment, Na_Ca treatment significantly reduced soluble sugar content by 39.11% (*p* < 0.05). These results suggest that exogenous calcium reduces salt stress damage to peanut roots by decreasing the content of soluble sugars, which maintains the osmotic pressure balance and reduces the degree of membrane lipid peroxidation.

Salt stress causes membrane lipid peroxidation, which may scavenge reactive oxygen species by enhancing antioxidant enzyme activity, thereby reducing damage caused by salt stress. Based on these findings, exogenous calcium may reduce the degree of salt stress-induced membrane lipid peroxidation.

### 3.2. Screening and Enrichment Analysis of Differentially Expressed Genes in Peanut Roots under Salt Stress by Exogenous Calcium

**Figure 2:**
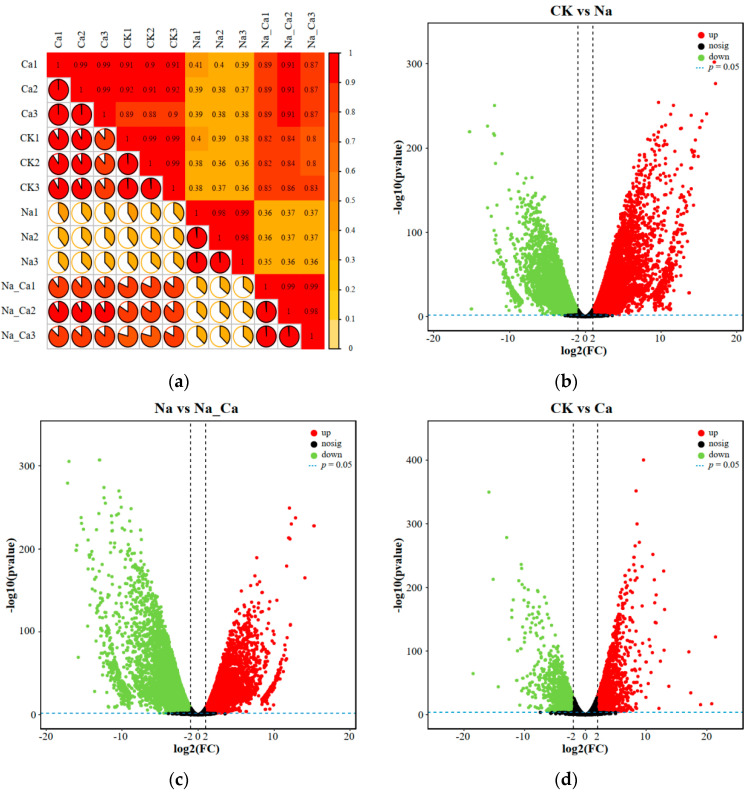
Preliminary analysis of transcriptomics data. (**a**) Correction heat map of different treatment groups. (**b**) Volcano plots of DEGs between CK and Na. (**c**) Volcano plots of DEGs between Na and Na_Ca. (**d**) Volcano plots of DEGs between CK and Ca.

RNA deep sequencing represents an alternative technological platform for studying transcriptional regulation, which can accurately determine which transcripts are present in a given sample. Gene expression can be calculated based on absolute transcript abundance. The previous data analyzed the effect of exogenous calcium on physiological indicators in peanut roots under salt stress. Based on these results, a transcriptome analysis was performed on peanut root systems 48 h after treatment. The Pearson correlation coefficients for each sample are shown in Figure 2a. The Pearson correlation coefficients between biological replicates under the same treatment were more significant than those between two samples under different treatments. As a result, the reproducibility of the samples under the same treatment is good, and the requirements of the downstream analysis can distinguish the differences between the treatment groups.

**Figure 3:**
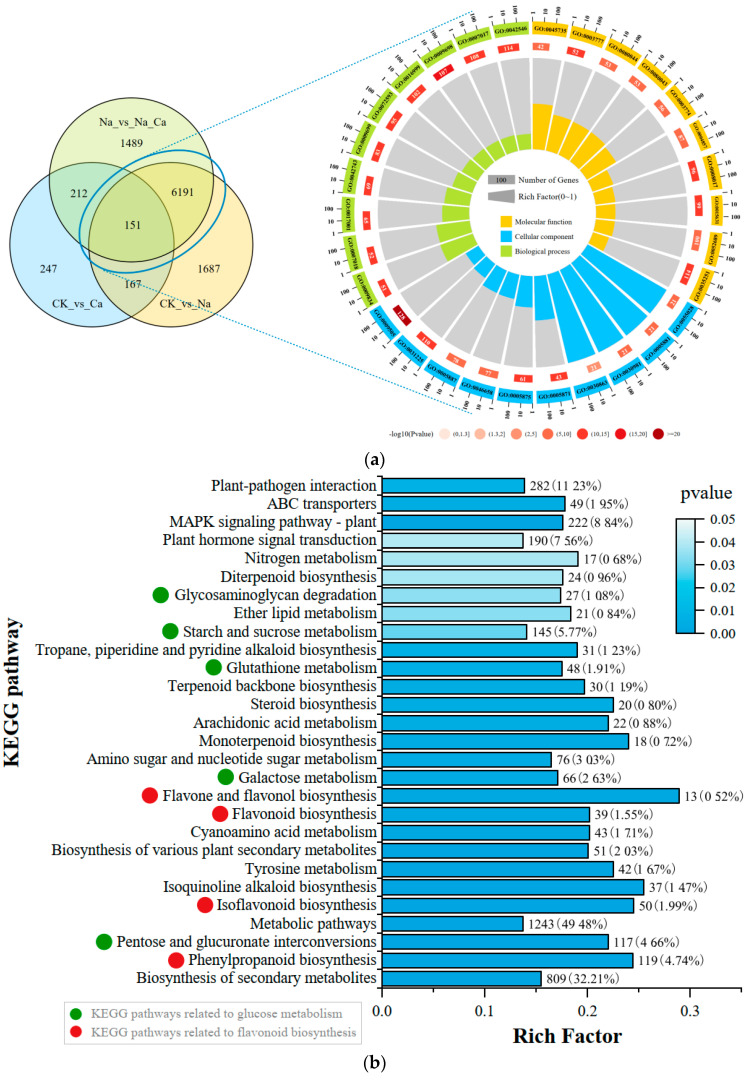
Venn diagram and enrichment analysis of DEGs. (**a**) Venn diagram of DEGs and circle plots of GO enrichment analysis. Three circles from inside out. First circle: enriched classifications; outside the circle is a sitting scale for the number of genes; different colors represent different classifications (yellow for molecular function, blue for cellular components, green for biological processes). Second circle: the number of background genes in that classification and the *p*-value. The more genes there are, the longer the bar, the smaller the value, and the darker the color. Third circle: the rich factor value of each classification (the number of foreground genes in that classification divided by the number of background genes), where each cell of the background auxiliary line represents 0.1. The results of the GO enrichment analysis were sorted according to the size of the P-value, and the top 30 results were selected and plotted in a circle graph. (**b**) KEGG enrichment analysis bar chart of DEGs (the length of the bar indicates the number of genes enriched into the pathway, and the darker the color, the smaller the *p*-value).

Volcano plots can visualize the distribution of differential genes among treatment and control groups. Figure 2b–d show the DEGs for the three comparison groups (CK vs. Na, Na vs. Na_Ca, and CK vs. Ca). There were 8196 DEGs between CK and Na, with 3935 genes up-regulated and 4261 genes down-regulated. Na and Na_Ca showed 8043 DEGs, with 3870 up-regulated and 4173 down-regulated. A total of 777 DEGs were present between CK and Ca, 420 of which were up-regulated, while 357 were down-regulated. Based on the Venn diagram analysis of the three comparison groups (Figure 3a), 6342 DEGs were found to be shared by CK vs. Na as well as Na vs. Na_Ca, which were considered to be differentially expressed genes “affected by salt stress and regulated by exogenous calcium under salt stress”. After GO enrichment analysis (Table S1), DEGs were enriched in molecular function, cellular composition, and biological process. According to the *p*-values of the GO terms, we ranked them in ascending order and plotted the top 30 GO terms in Figure 3a. Two GO terms were noted. One was associated with plant antioxidants, including reactive oxygen species metabolic process (GO:0072593) and hydrogen peroxide metabolic process (GO:0042743). The second category was associated with plant secondary metabolite biosynthesis: phenylpropanoid metabolic process (GO:0009698), quercetin 7-O-glucosyltransferase activity (GO:0080044), quercetin 3-O-glucosyltransferase activity (GO:0080043), and quercetin UDP-glucosyltransferase activity (GO:0035251). The hierarchical relationships of these six GO terms were also plotted (Figure S2).

Further, KEGG enrichment analysis was performed on the DEGs shared by “CK vs. Na” and “Na vs. Na_Ca” (Table S2), and 28 pathways with *p* < 0.05 were selected and plotted as bar graphs (Figure 3b). Differentially expressed genes were enriched in two classes of pathways (9 in total). Five DEGs were involved in sugar metabolism, including 27 DEGs involved in glycosaminoglycan degradation (ko00531), 145 DEGs involved in starch and sucrose metabolism (ko00500), 48 DEGs involved in glutathione metabolism (ko00480), 66 DEGs involved in galactose metabolism (ko00052), and 117 DEGs involved in pentose and glucuronate interconversions (ko00040). Four other pathways were related to flavonoid biosynthesis, including 119 DEGs enriched in phenylpropanoid biosynthesis (ko00940), 39 DEGs enriched in flavonoid biosynthesis (ko00941), 13 DEGs enriched in flavone and flavonol biosynthesis (ko00944), and 50 DEGs enriched in isoflavonoid biosynthesis (ko00943). The results of the GO enrichment analysis and KEGG enrichment analysis suggest biosynthesis-related pathways by modulating reactive oxygen species homeostasis in peanut roots under salt stress.

### 3.3. Regulation of Differentially Expressed Genes of Sugar Metabolism and AsA-GSH Cycle by Exogenous Calcium in Peanut Roots under Salt Stress

**Figure 4:**
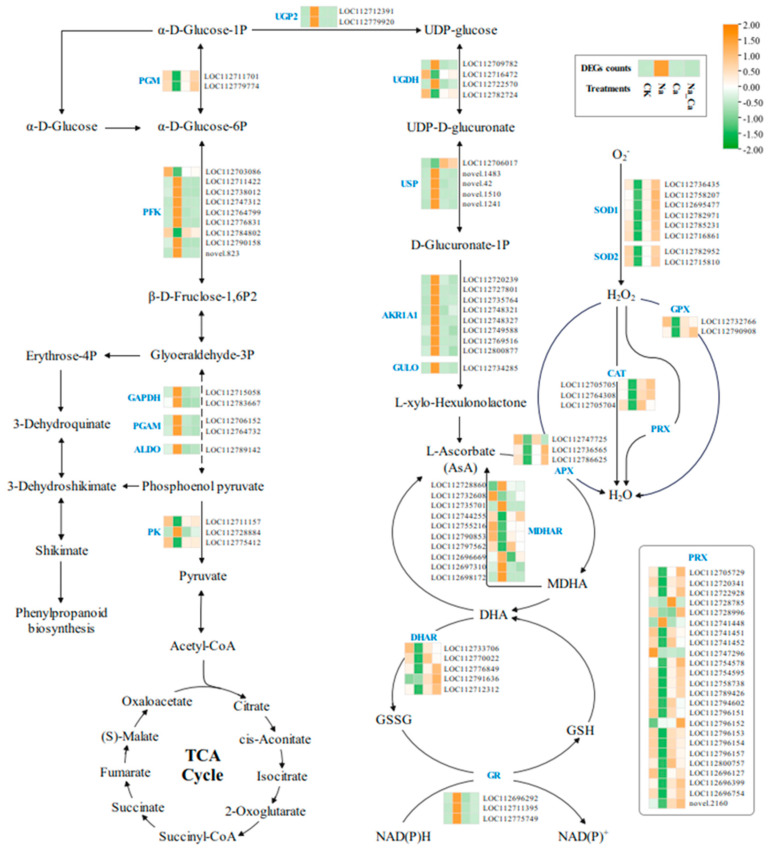
Heatmap of the pathway of glycolysis, antioxidant enzymes, and ascorbate–glutathione cycle. Pathways were constructed based on the KEGG pathway and references. The four treatments are CK, Na, Ca, and Na_Ca from left to right. The squares indicate the average expression of the genes corresponding to the key enzymes in the biosynthetic pathway under the four treatments, where orange indicates up-regulation and green indicates down-regulation.

The AsA-GSH cycle is a major enzymatic reaction pathway for eliminating reactive oxygen species. Physiological phenotyping in this study showed that exogenous calcium increased SOD, CAT, and POD activities and effectively reduced MDA content under salt stress. In addition, it inhibited the production of O^2−^ and H_2_O_2_. Moreover, the KEGG enrichment analysis also revealed that more DEGs were enriched in the sugar metabolism pathway. Therefore, we drew a gene expression pathway heat map (Figure 4) for the DEGs involved in sugar metabolism and the AsA-GSH cycle to investigate their expression patterns under different treatments.

The figure shows that the expression of key enzyme genes in both the glycolytic metabolic pathway and ascorbic acid biosynthesis pathway are significantly up-regulated under Na treatment compared to that with CK treatment. It was observed that the expression of key enzyme genes in the AsA-GSH cycle and antioxidant enzyme enzymatic systems showed a significantly down-regulated trend. In contrast, the activities of SOD, CAT, and POD (Figure 1) were consistent with the expression trend of key enzyme genes. Although the trend of Na_Ca treatment was opposite to that of Na treatment, key enzyme genes had a significantly down-regulated expression pattern in both the glycolytic metabolic and ascorbic acid biosynthesis pathways. In contrast, the expression patterns of key enzyme genes in both the AsA-GSH cycle and the antioxidant enzyme defense system were significantly up-regulated. In other words, exogenous calcium extensively up-regulated the genes encoding antioxidant enzymes in the AsA-GSH cycle and down-regulated the expression of key enzyme genes in sugar metabolism in peanut roots under salt stress.

### 3.4. Regulation of Flavonoid Biosynthesis by Exogenous Calcium in Peanut Roots under Salt Stress

**Figure 5:**
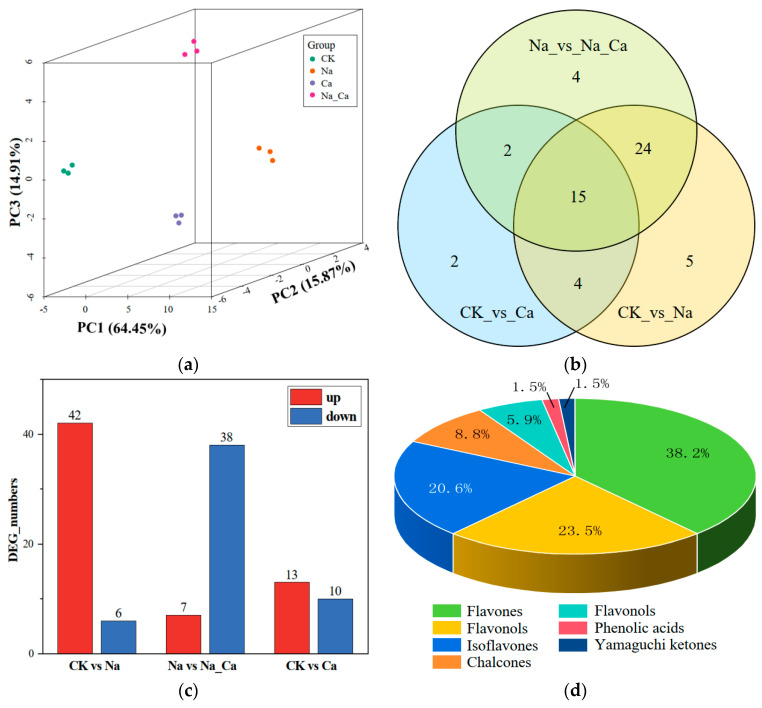
Preliminary analysis of metabolomics data. (**a**) Three-dimensional PCA score plots of metabolite profiles from different treatment groups. (**b**) Venn diagram of DAMs. (**c**) Histograms of up-and down-regulated flavonoids in three two-by-two comparison groups. (**d**) Three-dimensional pie chart of classification and proportion of 68 flavonoids detected in the peanut root.

**Figure 6:**
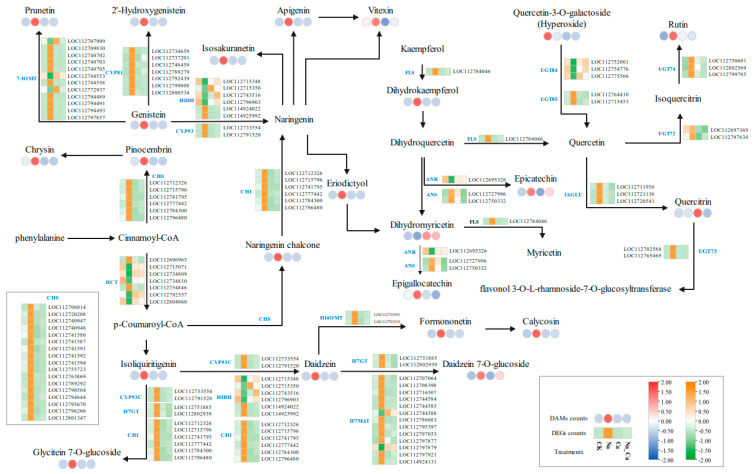
Heat map of the pathways of DEGs and DAMs in the flavonoid synthesis pathway under different treatments. Pathways were constructed based on the KEGG pathway and references. The four treatments are CK, Na, Ca, and Na_Ca from left to right. Data in boxes indicate the average expression of DAMs in the four treatments, indicated by circles, where red indicates up-regulation and blue indicates down-regulation. The square under the arrows indicates the average expression of genes corresponding to key enzymes in the biosynthetic pathway under the four treatments, where orange indicates up-regulation and green indicates down-regulation.

Compared with enzymatic systems, as essential antioxidants in non-enzymatic systems, the hydroxyl groups of flavonoids can also be used as a source of hydrogen to scavenge excessive reactive oxygen species. The results of GO enrichment analysis (Figure 3a) and KEGG enrichment analysis (Figure 3b) indicated that exogenous calcium led to differential expression of key enzyme genes in the massive flavonoid biosynthesis pathway in the peanut root system under salt stress. Therefore, we measured the absolute content of flavonoids in peanut roots under different treatments, screened for flavonoid metabolites that were differentially accumulated under salt stress regulated by exogenous calcium (Figure 5) and then mapped the pathway heatmap in conjunction with the differential expression of key enzyme genes (Figure 6), in order to investigate the regulation of the flavonoid biosynthesis pathway in peanut roots under salt stress by exogenous calcium.

#### 3.4.1. Effect of Exogenous Calcium on Absolute Flavonoid Content in Peanut Roots under Salt Stress

The absolute content of flavonoids chemically synthesized in the peanut root system under different treatments was determined by applying the UPLC-MS/MS technique, and 68 flavonoids were identified, which could be divided into seven subclasses (Figure 5d). Based on their percentages of species, they were flavones, flavonols, isoflavones, chalcones, flavonols, phenolic acids, and yamaguchin ketones, in order of their highest to the lowest percentages. The sum of the percentages of the three flavonoid subclasses of flavones, flavonols, and isoflavones was 82.3%. The projections of the individual samples on the first principal component, the second principal component, and the third principal component were analyzed using principal components and plotted on a three-dimensional scatter plot (Figure 5a). In 3D space, sample points were clearly separated between treatments, indicating a specific accumulation pattern for various flavonoids.

Volcano plots (Figure S3) were drawn for the differentially accumulated metabolites (DAMs) in each of the three two-by-two comparison groups (CK vs. Na, Na vs. Na_Ca, and CK vs. Ca). Based on the two-by-two comparison, the differential expression threshold was defined as |Log_2_FC| > 2 and *p* < 0.05. The up- and down-regulated DAMs in each comparison group were then calculated and plotted in grouped bar charts (Figure 5c). Salt stress up-regulated 42 flavonoid accumulations. In comparison, salt stress down-regulated six flavonoid accumulations. Salt stress resulted in the up-regulation of only seven flavonoid accumulations and the down-regulation of 38 flavonoid accumulations when exogenous calcium was administered. In the three comparison groups, adding exogenous calcium increased the accumulation of 13 flavonoids and decreased the accumulation of 10 flavonoids. Figure 2 shows the types and regulatory trends of the differentially accumulated flavonoids in the three comparison groups. A Venn diagram was then used to analyze the differential accumulation of flavonoids in the three comparison groups (Figure 5b). According to Figure 5b, 39 flavonoids shared between CK and Na and Na and Na_Ca were “affected by salt stress and regulated by exogenous calcium”. Table S3 illustrates their absolute contents under different treatment conditions.

#### 3.4.2. Regulation of Flavonoid Biosynthesis by Exogenous Calcium in Peanut Roots under Salt Stress

Among the 39 differentially accumulated flavonoids, 23 could be enriched in KEGG pathways related to flavonoid biosynthesis. Combined with the results of the KEGG enrichment analysis of differentially expressed genes in the transcriptome, we drew a heat map of flavonoid biosynthesis (Figure 6). We attempted to comprehensively investigate the regulation of peanut root flavonoid biosynthesis pathways under salt stress by exogenous calcium from the gene expression levels and metabolite biosynthesis.

In the flavonoid biosynthesis-related pathways, the majority of differentially expressed genes showed the same trend of changes in expression under different treatments. Na treatment significantly up-regulated their expression compared with CK treatment, while Na_Ca treatment significantly down-regulated their expression compared with Na treatment. However, a few differentially expressed genes showed opposite expression patterns under different treatment conditions. The following locations are included: three *UGT84* (LOC112752001, LOC112754776, and LOC112775566), four *HIDH* (LOC112715348, LOC112715350, LOC112743516, and LOC112796903), one *ANR* (LOC112695326), one *UGT73C* (LOC112754776), two *IF7MAT* (LOC112744588 and LOC112797879), and five *HCT* (LOC112715071, LOC112734809, LOC112734810, LOC112792557, and LOC112804060).

Among the 23 differentially accumulated flavonoid metabolites, except for Quercetin-3-O-galactoside (Hyperoside), Quercitrin, and Dihydromyricetin, the remaining 20 showed the same accumulation pattern under different treatments. Na treatment up-regulated their expression compared to CK treatment, whereas Na_Ca treatment reduced their expression compared to Na treatment. This is especially the case with flavonols and isoflavones and flavonoids, such as naringenin, chalcone, isoflavone, glycyrrhizin, soy glycosides, prickly awn stalked phloem, mullein, chrysanthemum, apigenin, prunetin, and so on. The results suggest that salt stress causes an increase in flavonoid accumulation and the expression of key enzyme genes in their biosynthetic pathways. At the same time, exogenous calcium negatively regulates flavonoids and key enzyme genes in their biosynthetic pathways.

#### 3.5. qRT-PCR Verification

**Figure 7:**
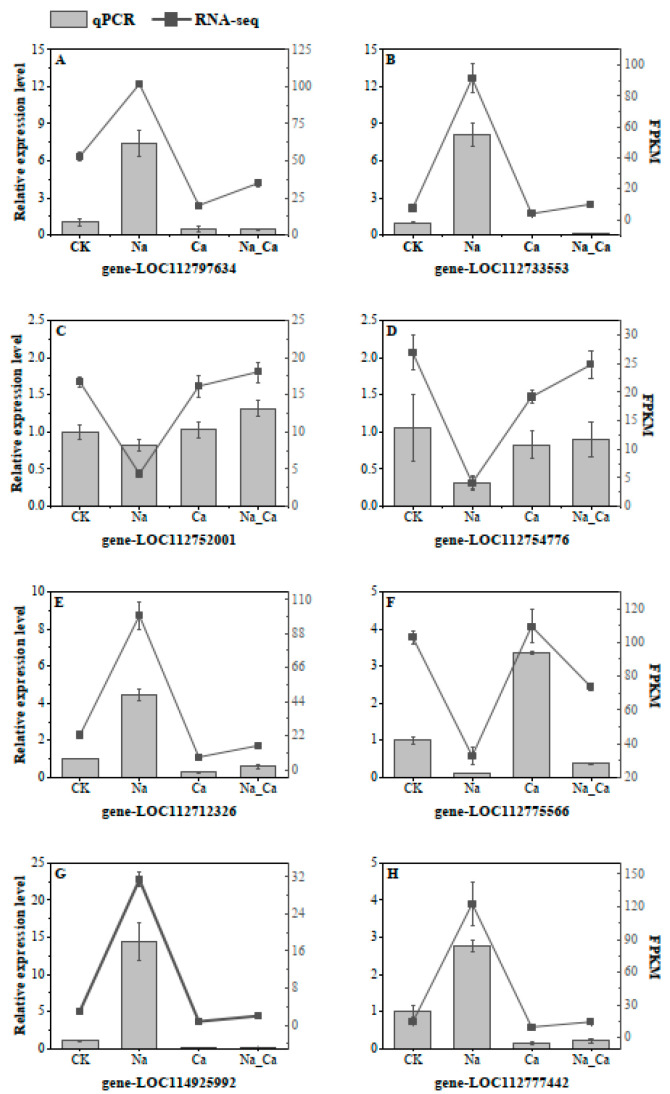
RT-qPCR was performed to verify the expression pattern of key enzyme genes involved in flavonoid synthesis in peanut roots under different treatments. Values presented are mean ± SD (n ≥ 3). (**A**–**H**) are the relative expression levels of 8 genes, and their abscissas are the corresponding genes. Bars represent the results of RT-qPCR, and lines represent the results of RNA-Seq. The scale on the left axis represents relative expression, and the right axis represents FPKM values.

The qRT-PCR experiments were conducted in both the control and treatment groups in order to verify the accuracy of the RNA-Seq data; eight DEGs in the roots of *Arachis hypogaea* L. were selected randomly. Figure 7 illustrates the reliability of the RNA-Seq results by demonstrating the same change trend for all eight genes.

## 4. Discussion

This study investigated how exogenous calcium and salt stress induce flavonoid synthesis and their roles in stress tolerance regarding gene transcription, enzymes, physiological indicators, and metabolites. We explored the antioxidant defense system in terms of primary and secondary metabolic pathways, enzymatic reactions, non-enzymatic reaction systems, and the AsA-GSH cycle in an integrated manner. As a protective mechanism against oxidative damage, the antioxidant defense mechanism reduces reactive oxygen species (ROS) production in plants during adverse conditions [37]. In this study, exogenous calcium had a good mitigating effect on the growth of peanut roots under salt stress. The addition of exogenous calcium alleviated the oxidative stress under adverse conditions and accelerated the elimination or detoxification of reactive oxygen species (ROS), mainly by increasing the activity of antioxidant enzymes and improving the efficiency of the ascorbic acid–glutathione cycle, as well as effectively reducing the MDA content. Zhang [38] investigated the effect of 20 μM melatonin on the salt tolerance of cotton. They found that POD activity and SOD activity were significantly increased in exogenous melatonin-treated cotton plants under salt stress. At the same time, we also found that the elevated antioxidant enzyme activities were accompanied by an up-regulation in the expression of the genes encoding the enzymes, as reported in our previous study, which was attributed to the significant up-regulation of differentially expressed genes encoding calcium-dependent protein kinases (CDPKs calcium-dependent protein kinases) after the addition of exogenous calcium [39], as Ca^2+^ receptor mediates rapid stress in plants in response to environmental changes [40], which in turn removes the excess reactive oxygen species in the cell to reduce oxidative stress under salt stress [24]. Mn-SOD overexpression in transgenic *Arabidopsis* plants increased salt tolerance [41], overexpression of Mn-SOD in transformed *L. esculentum* plants also increased salt tolerance [42], and transgenic tobacco plants overexpressing Cu/Zn-SOD also demonstrated increased tolerance to multiple stresses [43]. According to these results, exogenous chemicals can all alleviate oxidative stress to varying degrees under adverse stress conditions, and increasing antioxidant enzyme activity and up-regulation of the expression of their encoding genes enhanced plant tolerance to stress. The AsA-GSH cycle efficiency increased along with the activity of the four enzymes in this cycle, maintaining a high level of oxidative capacity.

Sugars can act as osmoregulators [44]. The emerging concept of “sugar as an antioxidant” has become increasingly evident, and sugar can also act as a true ROS scavenger in plants [19,45]. Also, soluble sugars accumulate under different biotic and abiotic stress conditions associated with oxidative stress, supporting the hypothesis that sugar is implicated in plant stress-induced ROS accumulation [46]. In this study, adding exogenous calcium effectively reduced the soluble sugar content under salt stress. It indicates that under salt stress, soluble sugars in the appropriate concentration range can enhance the plant’s ability to scavenge reactive oxygen species and protect the plant cells from oxidative damage [47], while the application of 15 mmol/L CaCl_2_ effectively mitigated the damage of salt stress on peanut roots, which we hypothesized might be since Ca^2+^ activated other antioxidant defense mechanisms in the plant, such as superoxide dismutase, superoxide dismutase, and calcium chloride. For example, SOD, APX, and other enzymatic systems are thought to play a significant role in the antioxidant defense system. It has been suggested that soluble sugars and enzymes in their glycolytic metabolic pathways are involved in oxidative stress and ROS signaling. However, their effects on gene expression are influenced by sugar-specific signaling cascades [46]. According to Koch [48], sugar variation can alter gene expression in abiotic stress-responsive enzymes, including SOD.

Additionally, sucrose has been shown to affect the biosynthesis and recycling of ascorbic acid (AsA) in broccoli florets [49]. Our findings are similar to this. This may be because, during oxidative stress, the protective effect of soluble sugars triggers direct or indirect signaling generated by ROS scavengers and repair enzymes [19]. Although hexokinase (HK), Snf1-related kinase 1, and INV have been identified as conserved sugar signaling components [50], their dual roles as nutrients and signaling molecules have hindered the accurate study of their associated mechanisms. In addition, sugar signaling is closely related to hormone signaling and stress-related pathways [51,52], complicating the antioxidant defense response to abiotic stress.

Flavonoids are a series of secondary metabolites produced downstream of the phenylpropane pathway [39] and a class of small molecules with antioxidant capacity formed in plants, which are closely related to plant resistance and can be used to scavenge ROS [38,53]. Our study found that the accumulation of flavonoids and the number of species increased under salt stress. In contrast, after adding exogenous calcium, the accumulation of flavonoids and the number of species significantly decreased. As a result of salt stress, we detected 63 flavonoids, while exogenous calcium added to salt stress resulted in 47 flavonoids being detected. Previous studies have shown that limes biosynthesize flavonoid compounds with enhanced antioxidant activity to detoxify the deleterious effects of reactive oxygen species produced during drought stress. Xiong et al. [54] showed that flavonoids can act as antioxidants and play a crucial role in maintaining cellular function under magnesium deficiency stress. Xu et al. [55] found that powdery mildew infection induced an accumulation of total flavonoid content in wheat, and exogenous flavonoid treatment of infected plants reduced the severity of infection. Flavonoids confer resistance to wheat powdery mildew by modulating the redox system, mainly by decreasing POD and CAT activities to maintain the balance of ROS and homeostasis, thus supporting the defense-related signaling cascade.

Furthermore, it has been reported that the total flavonoid content of sorghum increased under moderate and severe salt stress with significant changes in the related synthesized genes [1]. High salt stress significantly increased the expression of flavonoid-synthesizing genes *DFR* and *ANS* in rice [56]. Integrated transcriptomic and metabolomic analyses reveal that flavonoids are essential in resisting powdery mildew. The contents of some flavonoids increased significantly after 24 h of exposure to NaCl. Further, after 1 or 6 h of NaCl treatment, the flavonoid synthesis genes *CHS*, *CHI*, *FLS*, and *ANS* were significantly up-regulated. The results of Ithal and our study are consistent with each other. In Arabidopsis, overexpression of the *CHS* gene significantly increased the amount of flavonoids, thus improving salt resistance [57]. When the *F3H* gene is over-expressed, the content of flavan-3-ols in tobacco plants (catechins and epicatechins) increases, and the antioxidant activity of tobacco plants is directly enhanced [58]. The total flavonoid content of tobacco that overexpressed the *FLS* gene was 1.84 times higher, and salt tolerance was higher than that of tobacco that did not overexpress the *FLS* gene [59]. Previous studies suggest that up-regulation of some genes involved in flavonoid synthesis, individually or in combination, can increase a plant’s flavonoid content, enhancing its ability to cope with stress. Moreover, other studies have demonstrated that flavonoids can enhance plant tolerance to abiotic stresses through direct or indirect modulation of oxygen-containing free radicals, peroxides, and reactive oxygen species. We found that NaCl stress significantly altered the expression levels of genes such as *CHS*, *CHI*, *FLS*, and *ANS* in peanut roots, which were then significantly down-regulated by exogenous calcium. According to the results of the present study, flavonoid synthesis is an important mechanism for adaptation to salt stress in peanuts. As a result of drought stress, Sperdouli and Moustakas [60] found an increased accumulation of flavonoids and soluble sugars in Arabidopsis thaliana leaves, maintaining a high level of antioxidant protection. Our findings are similar to these. In addition, we hypothesized that the glycolytic metabolic pathway might be a bridge connecting the phenylpropane and its flavonoid biosynthesis pathway to antioxidant systems such as the AsA-GSH and that the glycolytic metabolic pathway became more active under adversity stress, producing more primary metabolites, which in turn allocated these excess metabolites to secondary metabolic pathways, such as the phenylpropane pathway and its flavonoid biosynthesis pathway, to continue synthesizing metabolites. Exogenous calcium was found to up-regulate the activity of the antioxidant enzymes of the antioxidant defense system and to activate the antioxidant capacity of the AsA-GSH cycle, which can be considered a significant component of the antioxidant defense system. At this time, the glycolytic pathway was operating normally. The metabolites were directly allocated to the TCA cycle, and the flavonoid biosynthesis substrates were lacking, so the amount and types of accumulations were reduced accordingly.

## 5. Conclusions

Based on our data, it can be demonstrated that enzymatic and non-enzymatic reaction systems have a reciprocal relationship, acting together in synergy as antioxidant protectors, with glycolysis acting as a bridge between antioxidant enzymes and the ascorbate–glutathione cyclic system and flavonoid biosynthesis. In this experiment, analysis of physiological data, transcriptomics, and absolute quantitative metabolomics of flavonoids showed that salt stress reduced root vigor and antioxidant enzyme activities in peanuts. In contrast, the expression of genes encoding antioxidant enzymes was significantly down-regulated. The expression of key enzyme genes in the ascorbate–glutathione cycle was significantly down-regulated. The content of superoxide anion and hydrogen peroxide surged. Glycolytic metabolic pathway and key enzyme genes in the flavonoid biosynthesis pathway were significantly up-regulated, and the accumulation of most flavonoids increased. In response to exogenous calcium, the balance between enzymatic and non-enzymatic systems was altered, leading to a more significant role for the enzymatic system and a diminished antioxidant effect for the non-enzymatic system. Both antioxidant defense systems work synergistically to alleviate salt stress-related damage. Ultimately, these studies will better understand how plants defend themselves against adverse conditions through antioxidant defense systems.

## Data Availability

The data presented in the study are deposited in the Sequence Read Archive (SRA) database at NCBI (SRA Bio Project PRJNA964590). Other study data can be found in the manuscript or Supplementary Materials.

## Supplementary Materials

The following supporting information can be downloaded at: https://www.mdpi.com/article/10.3390/antiox13020233/s1, Figure S1: An overview of the experimental data analysis workflow; Figure S2: Hierarchy of six GO terms; Figure S3: Flavonoid DAMs Volcano Plots in three pairwise comparison groups; Figure S4: Flavonoid DAMs regulation patterns in three pairwise comparison groups; Table S1: Results of GO enrichment in DEGs; Table S2: Results of KEGG enrichment in DEGs; Table S3: The contents of 39 flavonoids were determined by UPLC-MS/MS; Table S4: Primer sequences of differentially expressed genes (DEGs) of RT-qPCR; Table S5: The expression of DEGs under different treatments in the heat map of flavonoid biosynthesis pathway.
