# Supplementary figures and images for: Exogenous Calcium Alleviates Oxidative Stress Caused by Salt Stress in Peanut Seedling Roots by Regulating the Antioxidant Enzyme System and Flavonoid Biosynthesis
Source: Antioxidants (Basel). 2024 Feb 14;13(2):233. doi: 10.3390/antiox13020233 (PMC10886236; doi:10.3390/antiox13020233)

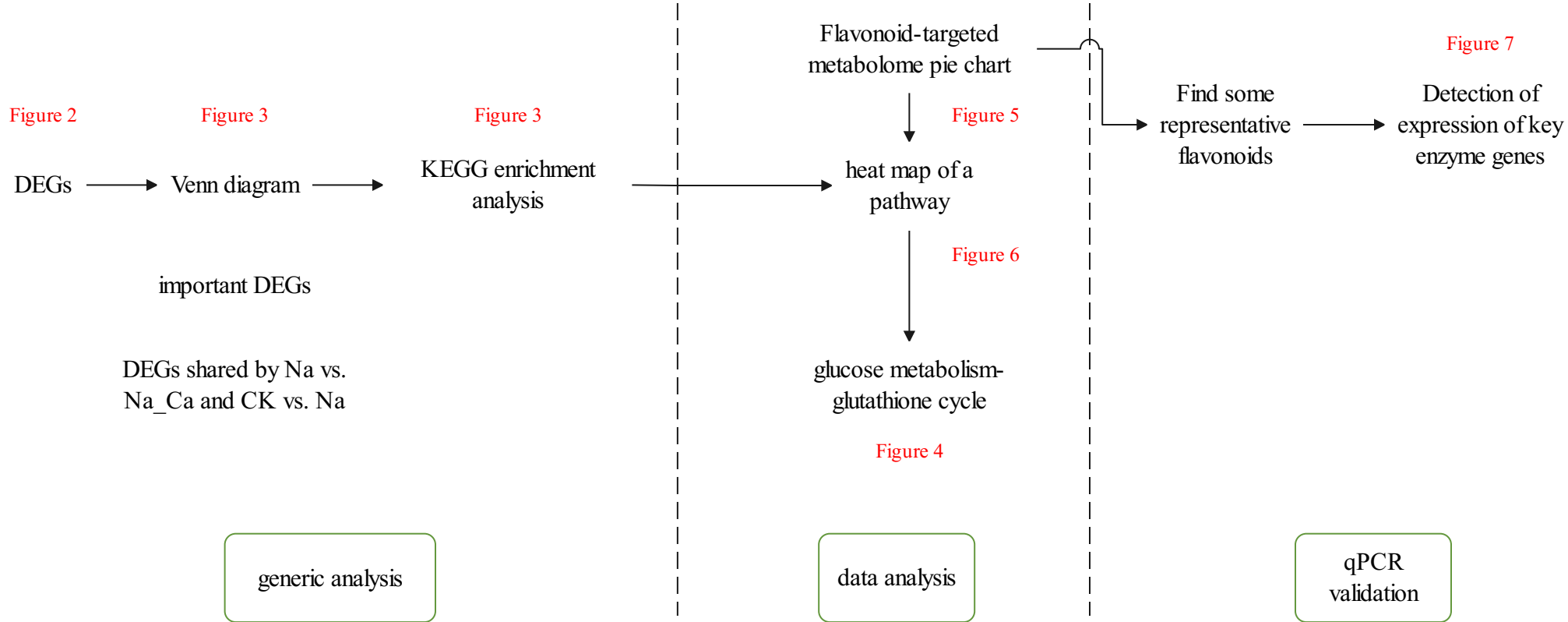

Figure S1. An overview of the experimental data analysis workflow

Supplement: Supplementary file 1 [file antioxidants-13-00233-s001.zip › Figure S1.pdf]

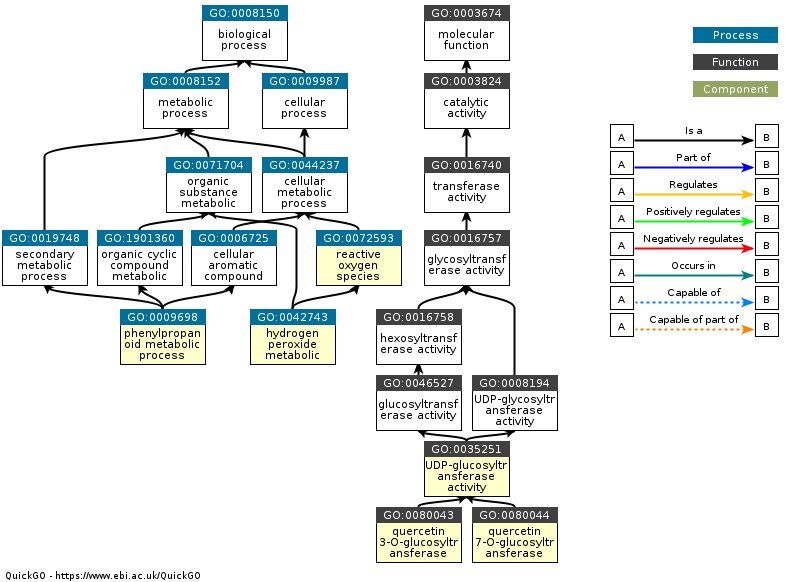

Supplement: Supplementary file 1 [file antioxidants-13-00233-s001.zip › Figure S2.jpg]

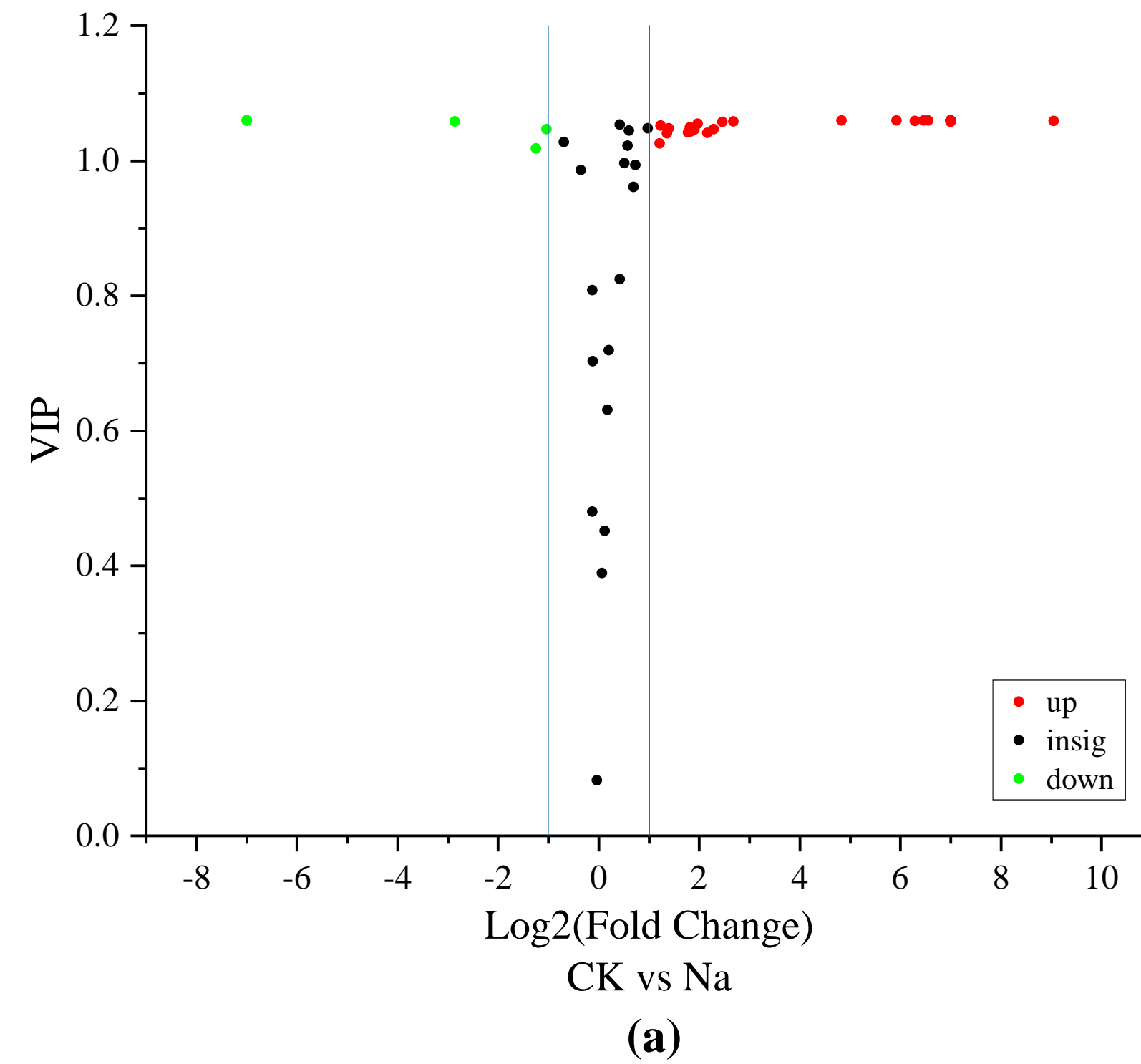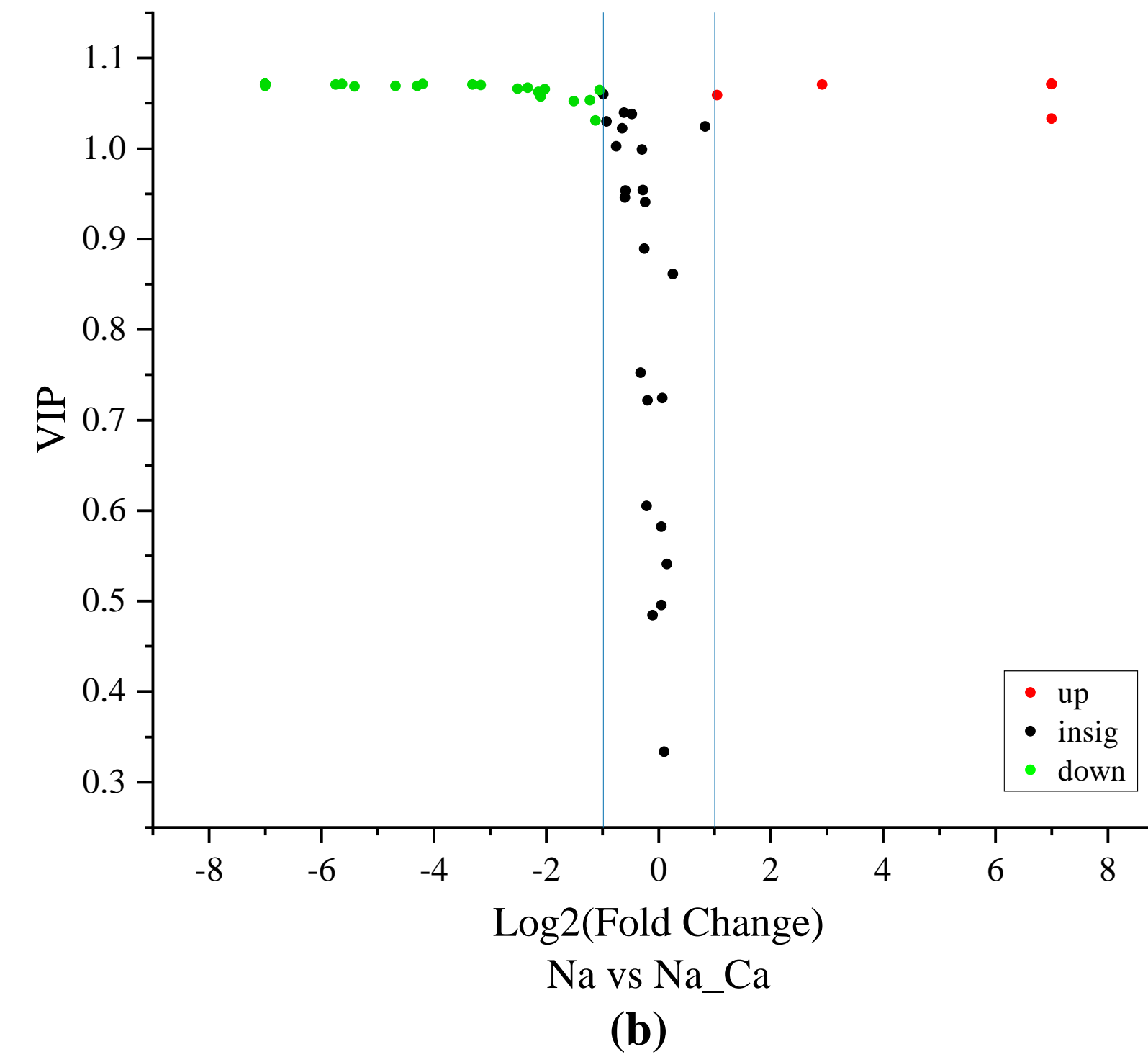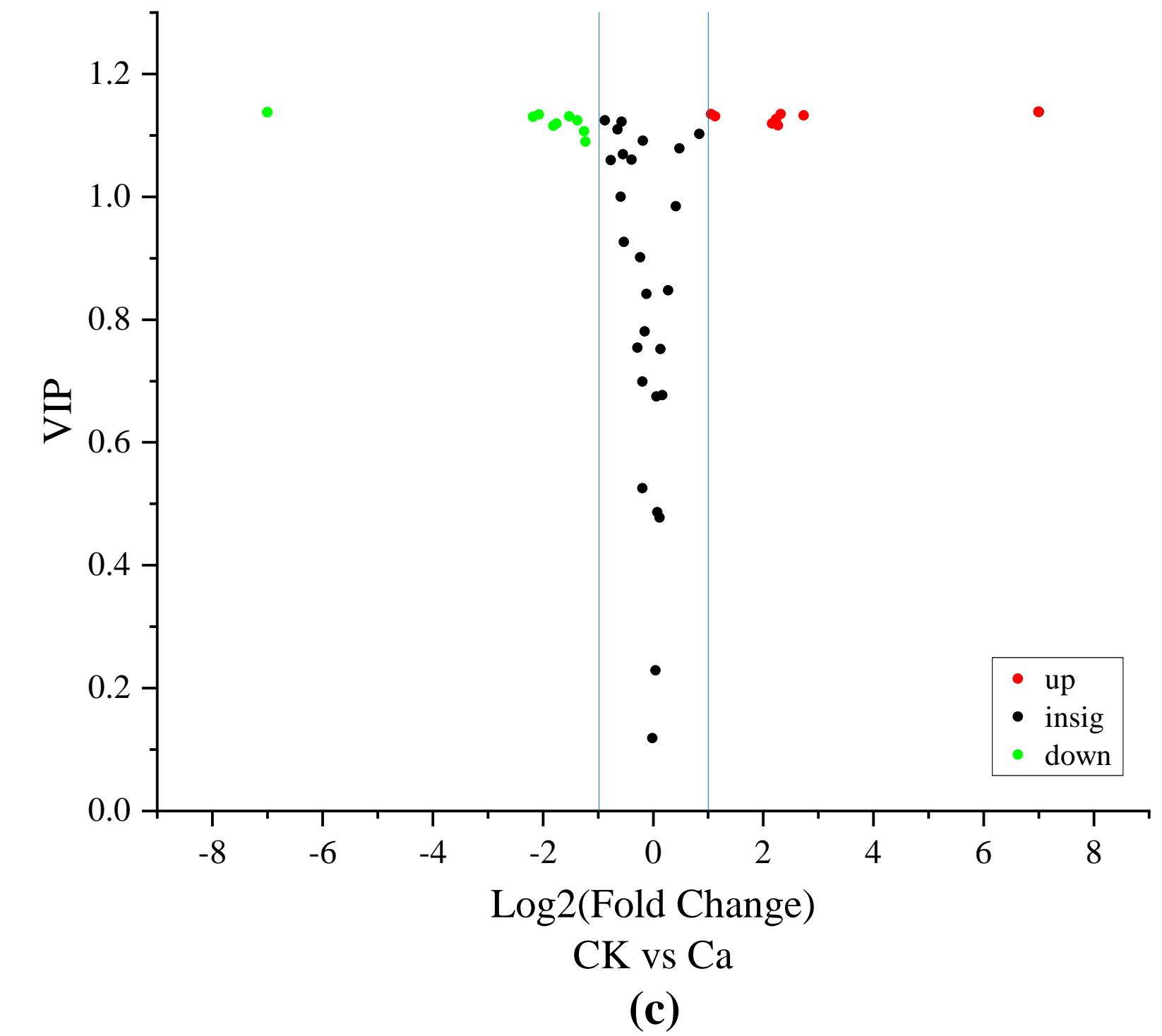

**Figure S3 Flavonoid DAMs Volcano Plots in three pairwise comparison groups.**

Supplement: Supplementary file 1 [file antioxidants-13-00233-s001.zip › Figure S3.pdf]

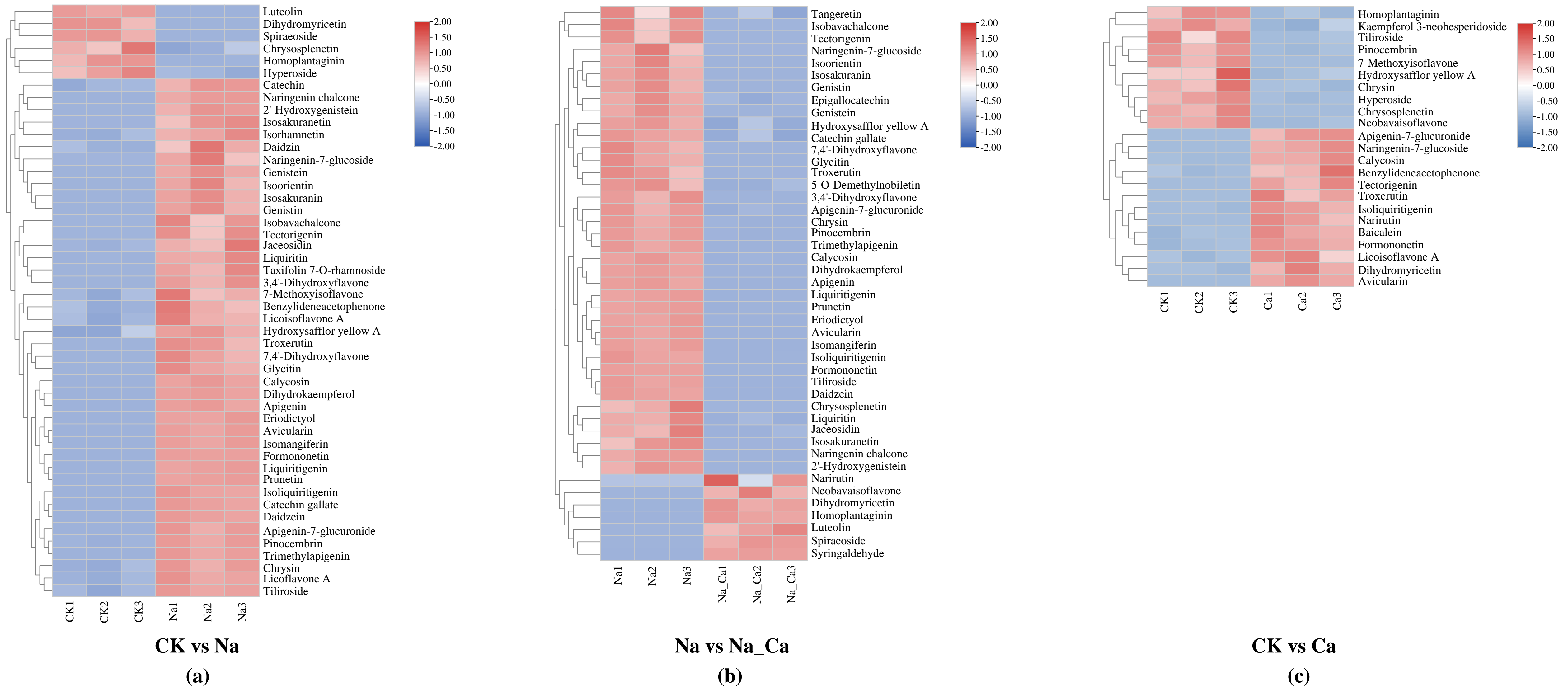

**Figure S4 Flavonoid DAMs regulation patterns in three pairwise comparison groups.**

Supplement: Supplementary file 1 [file antioxidants-13-00233-s001.zip › Figure S4.pdf]
